# Evading native restriction-modification systems improves electroporation efficiency of the methanotroph *Methylococcus capsulatus* Bath

**DOI:** 10.1128/aem.00538-26

**Published:** 2026-06-12

**Authors:** Yao-Chuan Yu, Melissa Tumen-Velasquez, Michael Melesse Vergara, William Alexander, Daniel Grinffiel, Jessica M. Henard, Adam M. Guss, Calvin A. Henard

**Affiliations:** 1 Department of Biological Sciences, BioDiscovery Institute, and Advanced Environmental Research Institute, University of North Texas 3404, Denton, Texas, USA; 2 Biosciences Division, Oak Ridge National Laboratory 551173, Oak Ridge, Tennessee, USA; Kyoto University, Kyoto, Japan

**Keywords:** methane bioconversion, methanotroph, electroporation, restriction-modification system, methyltransferase, synthetic biology, CRISPRi, biomanufacturing

## Abstract

*Methylococcus capsulatus* Bath is a unique methanotrophic bacterium that uses methane (CH_4_) as a carbon and energy source and encodes the RubisCO enzyme for inorganic carbon assimilation, positioning it as an excellent candidate for biotechnology to capture atmospheric CH_4_ and carbon dioxide (CO_2_) for chemical production. However, a limited genetic toolbox and minimal high-throughput methodologies for genetic engineering hinder the development of efficient *M. capsulatus* biocatalysts. In this work, we developed and optimized an electroporation workflow to transfer circular and linear DNA fragments into *M. capsulatus* with high efficiency by evading the host restriction-modification (RM) systems that recognize and degrade foreign DNA. To achieve this, we determined the *M. capsulatus* methylome to identify RM system methyltransferases and express them in *Escherichia coli*, resulting in DNA methylation in *E. coli* that mirrors that of *M. capsulatus*, which improved methanotroph electroporation efficiency. Iterative site-directed mutagenesis of plasmid methylation sites identified a m^6^A motif necessary for the increase in electroporation efficiency. An *M. capsulatus* knock-out strain with a disrupted Type I RM system linked to the m^6^A motif showed improved transformation efficiency compared to wild type, underscoring the importance of proper m^6^A methylation in successful DNA transfer to *M. capsulatus*. Notably, plasmid methylation coupled to an optimized electroporation workflow enabled the facile generation of an *M. capsulatus* genome-wide CRISPR interference library for functional genomic screening. Collectively, our results identify an RM system that has, up to now, limited *M. capsulatus* transformation and establish a methodology to bypass the RM system and improve transformation efficiency, which will accelerate the development of engineered methanotrophs for CH_4_ bioconversion technologies.

**IMPORTANCE:**

Aerobic CH_4_-oxidizing bacteria (methanotrophs) utilize CH_4_ as a carbon and energy source in nature, also making them promising biocatalysts for converting CH_4_ to valuable products. However, the development of these non-model microbes for use in biotechnology has been slowed by limitations in genetic tools and methodologies. Here, we established DNA electroporation in the methanotroph, *Methylococcus capsulatus* Bath, which had poor baseline DNA transfer efficiency. By determining the *M. capsulatus* methylome and identifying its restriction-modification systems that digest foreign DNA, thereby limiting DNA transformation, we developed an *Escherichia coli* cloning strain expressing *M. capsulatus* methyltransferases that modify plasmid DNA mirroring the host, significantly improving DNA transfer to *M. capsulatus*. These improvements enabled the proof-of-concept construction of a genetic library via electroporation. The significant advancements herein bring us closer to realizing the use of methanotrophs for biomanufacturing and atmospheric CH_4_-mitigating biotechnologies.

## INTRODUCTION

Aerobic methane (CH_4_)-oxidizing bacteria (methanotrophs) utilize CH_4_ as their carbon and energy source and thus represent a microbial chassis for simultaneous mitigation of greenhouse gas (GHG) emissions and conversion of CH_4_ to valuable bioproducts (1). Indeed, these bacteria have been engineered to convert CH_4_ to an array of products (2–6), and the model methanotroph, *Methylococcus capsulatus* Bath, is currently used industrially for the production of single-cell protein (7–9). Notably, *M. capsulatus* is a unique methanotroph that encodes the ribulose-1,5-bisphosphate carboxylase/oxygenase (RubisCO) enzyme and assimilates CO_2_ in addition to CH_4_, making it an excellent methanotroph candidate for the development of CH_4_ and CO_2_ conversion biotechnologies (10–12).

Several tools and methodologies have been developed for genetic modification of methanotrophs, including a suite of broad-host-range plasmids, regulatory promoter elements, and a CRISPR system for genome editing (13–15). Plasmids are routinely transferred to methanotrophs via conjugation with efficiencies ranging from 10^1^ to 10^4^ transconjugants per mating (16). Electroporation methods for DNA transfer have also been developed for a subset of methanotrophs, including the Alphaproteobacterial methanotrophs *Methylocystis* sp. SC2, *Methylocella silvestris* BL2, and *Methylosinus trichosporium* OB3b (17–19), and the Gammaproteobacterial methanotrophs *Methylotuvimicrobium buryatense, Methylomonas* sp*.*, *Methylobacter tundripaludum,* and *Methylotuvimicrobium alcaliphilum* (20–24); but the transformation efficiencies are lower than conjugation-mediated DNA transfer. In *Methylotuvimicrobium buryatense*, successful plasmid transformation via electroporation was achieved only when the plasmid was derived from the methanotroph rather than *Escherichia coli* (20), suggesting methanotroph restriction-modification (RM) systems may limit transformation of DNA isolated from *E. coli*. Although DNA can be transferred to facilitate plasmid-based protein expression and the generation of knock-out strains in several methanotrophs (15, 19, 25), DNA transformation efficiency improvements are needed to improve consistency and enable the development of the high-throughput genetic engineering methodologies required to realize the use of methanotrophs in biomanufacturing.

RM systems are encoded by most bacteria and function as a defense mechanism against foreign DNA (26, 27). They typically comprise a restriction endonuclease (REase) that recognizes and cleaves a specific target DNA sequence and a cognate methyltransferase (MTase) that modifies adenine or cytosine bases within the REase recognition sequence, preventing cleavage. Foreign DNA lacking host methylation patterns is subject to restriction, allowing discrimination of self from non-self DNA. Furthermore, the motifs targeted by RM systems are hypervariable, even between very closely related strains. Consequently, RM systems can be a significant barrier in bacterial genetic and metabolic engineering, where successful DNA transformation is requisite (20, 28).

One strategy to neutralize RM systems as a barrier to genetic transformation is to methylate plasmid DNA in *E. coli* prior to transformation into the target organism. Methylome analysis, for instance, using Oxford Nanopore Technologies (ONT) sequencing with MIJAMP motif analysis (29), can be used to determine the DNA sequence(s) that are methylated in the target organism, and expression of the corresponding DNA methyltransferases in *E. coli* can methylate and protect plasmid DNA prior to transformation (30–33). This approach has been used to increase the transformation efficiencies of several industrially promising microbial chassis, including *Clostridium thermocellum*, *Methylomonas* sp. DH-1, and *Synechococcus* sp. pCC7002 (23, 32, 34).

In this work, we show that electroporation methods previously used in other methanotrophs can also successfully transfer circular and linear DNA to *M. capsulatus*, albeit with low efficiency. To overcome the low efficiency, we determined the *M. capsulatus* methylome via ONT sequencing and engineered an *E. coli* strain to express heterologous *M. capsulatus* methyltransferases to mirror the methanotroph DNA methylation. Plasmid DNA isolated from the engineered *E. coli* strain showed significant improvements in transformation efficiency. Site-directed mutagenesis of putative methylation sites on the plasmid led to the identification of an m^6^A motif as necessary and sufficient for improved transformation efficiency. We generated an *M. capsulatus* knockout strain lacking a putative Type I RM system restriction enzyme hypothesized to recognize unmethylated m^6^A motifs, which showed improved transformation with DNA lacking host methylation patterns. Lastly, leveraging the methanotroph methyltransferase-expressing *E. coli* cloning strain and electroporation methodologies developed herein, we constructed a genome-wide CRISPR interference (CRISPRi) library consisting of ~1.4 × 10^4^ unique sgRNAs targeting all open reading frames in the *M. capsulatus* genome. Collectively, these findings significantly advance the methanotroph genetic toolbox and demonstrate methodologies for high-throughput genetic and metabolic engineering of methanotrophs.

## RESULTS AND DISCUSSION

### Plasmid and linear DNA can be successfully transferred to *M. capsulatus* via electroporation

**Fig 1:**
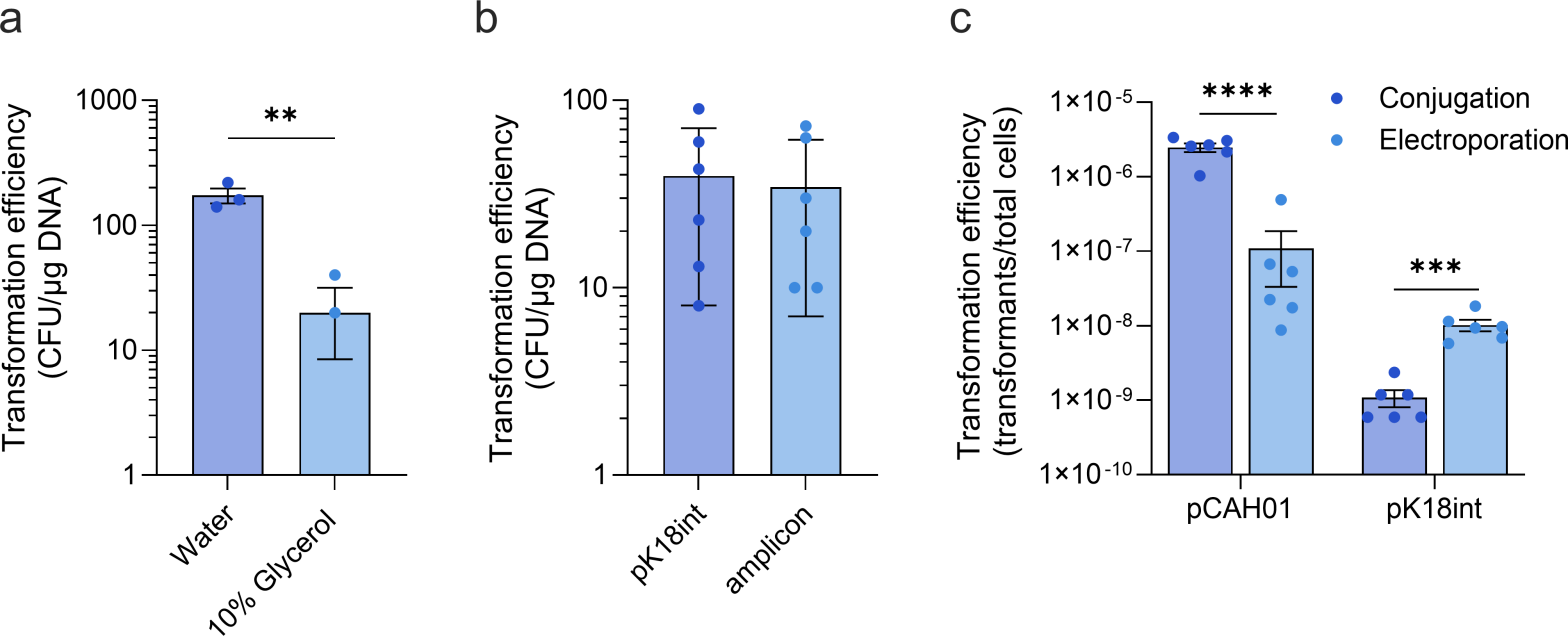
*M. capsulatus* Bath baseline electroporation efficiency. (**a**) Transformation efficiency of the IncP-based broad-host-range replicative plasmid pCAH01 via electroporation into *M. capsulatus* cells washed with either water or 10% glycerol as the wash buffer during electrocompetent cells preparation. (**b**) Transformation efficiency of the suicide plasmid pK18int and a linear marker-exchange mutagenesis cassette PCR amplified from pK18int as a template. (**c**) Conjugation and electroporation transformation efficiencies of replicative plasmid pCAH01 vs integration of pK18int into the *M. capsulatus* chromosome. The data represent the mean ± SEM from two independent experiments (*n* = 3–6). **P ≤ 0.01, ***P ≤ 0.001, ****P ≤ 0.0001.

Biparental or triparental conjugation using donor *Escherichia coli* strains is commonly used for DNA transfer to *M. capsulatus* (25, 35), which limits transferable DNA to plasmids. Electroporation is an alternative DNA transformation approach that enables direct transfer of both linear and circular DNA and requires fewer steps than conjugation (36, 37), but has only been successfully applied to a few methanotrophs (20, 22), not including *M. capsulatus*. In contrast to typical electrocompetent cell preparation, wherein cells are washed with osmoprotectants like 10% glycerol (37), past electroporation of methanotrophs has been enabled or improved by washing the cells with water (20, 22). We therefore tested whether electroporation could be used to transform *M. capsulatus*, either with water or glycerol as the wash buffer. Electroporation was achieved in both cases, with higher transformation efficiency of the IncP-based broad-host-range replicative plasmid pCAH01 using cells prepared with ddH_2_O (173 CFU/μg DNA) compared with those washed with 10% glycerol (20 CFU/μg DNA) (Fig. 1a).

We also evaluated the electroporation efficiency of marker-exchange mutagenesis by comparing recombination of linear or plasmid DNA via integration of a gentamicin resistance cassette into an intergenic region of the *M. capsulatus* chromosome. Electroporation of the control pK18mobpheS (38)-derived suicide plasmid, pK18int, yielded few gentamicin-resistant transformants (35–40 CFU/μg DNA). A similar number of colonies was obtained using a linear marker-exchange cassette that was PCR-amplified from pK18int (Fig. 1b). Colony PCR using primers flanking the integration site confirmed chromosomal integration into the expected locus (data not shown). This successful integration of linear DNA can significantly decrease the cloning and conjugation time required for generating *M. capsulatus* knock-out or knock-in strains and enables methodologies that have been employed in *Methylotuvimicrobium* spp. (20, 21) to be applied to *M. capsulatus*.

For a quantitative comparison between electroporation and conjugation transformation efficiencies, we normalized the electroporation transformant and transconjugant CFUs to the amount of methanotroph cells electroporated or mixed with the S17-1λ*pir* donor *E. coli*, respectively. As shown in Fig. 1c, electroporation is less efficient than conjugation when transferring a replicative plasmid (1.1 × 10^−7^ vs 2.5 × 10^−6^). In contrast, DNA delivery via electroporation seems to promote homologous recombination after transferring the pK18int suicide plasmid compared to transfer via conjugation (1 × 10^−8^ vs 1.1 × 10^−9^, Fig. 1c).

**Fig 2:**
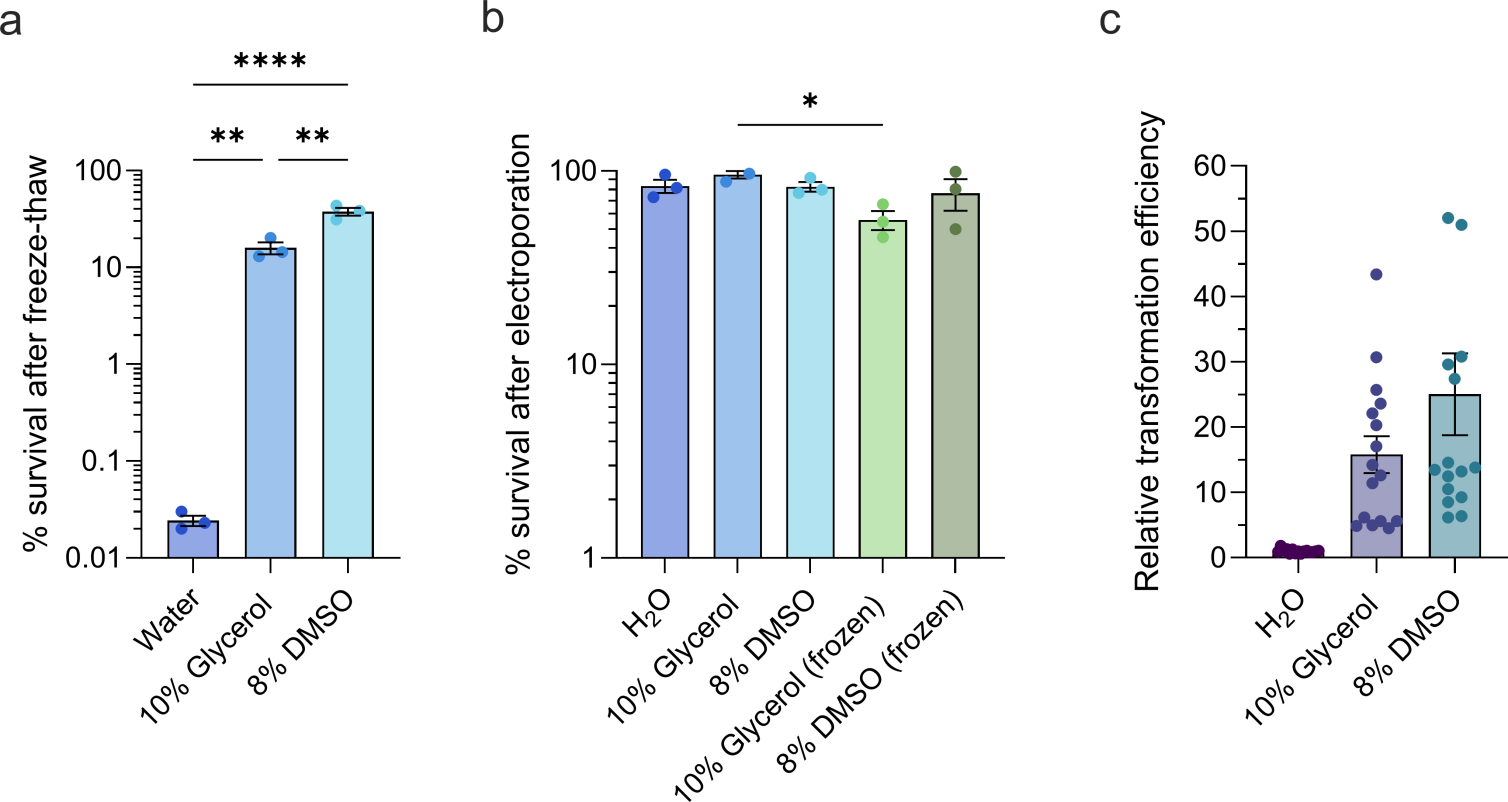
The inability to cryopreserve *M. capsulatus* electrocompetent cells is not due to cell death after freeze-thaw. (**a**) Percent survival of *M. capsulatus* after storage for 1 week at −80°C in water with or without 10% glycerol or 8% DMSO as a cryoprotectant and thawing on ice. (**b**) The effect of freeze-thaw, followed by electroporation, on *M. capsulatus* cell viability compared to electrocompetent cells freshly prepared with water, followed by the addition of 10% glycerol or 8% DMSO prior to electroporation. (**c**) Relative transformation efficiency of pCAH01 into cells washed with water, followed by the addition of 10% glycerol or 8% DMSO prior to electroporation. The data represent the mean ± SEM from at least two independent experiments (*n* = 3–14). *P ≤ 0.05, **P ≤ 0.01, ****P ≤ 0.0001.

We next determined whether the *M. capsulatus* electrocompetent cells could be cryopreserved at −80°C. Unfortunately, we did not obtain any transformants using previously frozen electrocompetent cells (data not shown). Therefore, we compared the effect of freezing with glycerol or DMSO cryoprotectants on cell survival and electroporation efficiency following freeze-thaw. Compared to the viability of the same batch of washed cells prior to freezing, 15% and 38% (equivalent to 1.1 × 10^10^ CFU/mL or 3.8 × 10^10^ CFU/mL) of cells remained viable after 7 days of being frozen at −80°C with 10% glycerol or 8% DMSO cryoprotectants, respectively, suggesting that the failure to transform plasmid DNA was not solely due to a decrease in cell viability (Fig. 2a). We also tested the possibility that previously frozen cells were more vulnerable to electrical shock, but the percentage of surviving cells between fresh and previously frozen cells following electroporation was comparable, except for those stored in 10% glycerol, which showed a slight, though statistically significant (*P* < 0.05), decrease in survival compared to fresh cells (Fig. 2b). Notably, the presence of 10% glycerol or 8% DMSO in the electroporation buffer did not decrease, and in many trials increased, transformation efficiencies (Fig. 2c). The underlying mechanism for the inability to transform previously frozen electrocompetent *M. capsulatus* cells is unclear, but a similar phenotype was also observed using previously frozen electrocompetent *M. buryatense* (20). It is possible that the freeze-thaw process induces physiological and/or structural changes that limit DNA transfer, but additional inquiry is needed to understand this phenotype.

### Improved electroporation efficiency by *in vivo* plasmid methylation in *E. coli* by *M. capsulatus* methyltransferases

Given our low electroporation efficiencies and the ubiquity of RM systems across bacteria, we hypothesized that native *M. capsulatus* RM systems could limit transformation efficiency by cleaving improperly methylated DNA derived from *E. coli*. We therefore performed methylome analysis upon this strain to identify DNA sequence motifs that might be cut by REases. To accomplish this, we sequenced the genome of our *M. capsulatus* strain using ONT and identified methylated DNA motifs using the MIJAMP software package (29). We identified six methylated motifs, including two motifs consistent with Type I RM systems [CCCm^6^A(N)_6_RTC/Gm^6^AY(N)_6_TGGG and Cm^6^AG(N)_6_CGTC/Gm^6^ACG(N)_6_CTG], two palindromic motifs consistent with Type II systems (RGm^6^ATCY and Gm^5^CGCGC), and one motif that could be Type II or III (CAAm^4^CTB) (Table 1).

**TABLE 1:** Methylation motifs identified in *M. capsulatus* and the *E. coli* AG15194 cloning strain that expresses *M. capsulatus* methyltransferases

Motif	*Methylococcus capsulatus* Bath (826 Mbp, 250X)^*a*^		*E. coli* methylation strain AG15194 (894 Mbp, 195X)^*a*^	
	% Modified	Count	% Modified	Count
RGm^6^ATCY	99.98	5,438	N/A^*c*^	N/A^*c*^
Gm^6^ATC	N/A^*c*^	N/A^*c*^	97.51	38,104
CCCm^6^A(N)_6_RTC^*b*^	92.86	588	0	595
Gm^6^AY(N)_6_TGGG^*b*^	99.83	588	13.1	595
Cm^6^AG(N)_6_CGTC^*b*^	88.99	772	94.2	779
Gm^6^ACG(N)_6_CTG^*b*^	99.87	772	94.9	779
Gm^5^CGCGC	96.36	5,410	99	4,302
CAAm^4^CTB	96.68	2,588	0	6,941

^*a*^ Amount of DNA sequence used in the analysis and fold-coverage of the genome.

^*b*^ Pairs of reverse complement sequences.

^*c*^ N/A, not applicable.

We next identified putative DNA methyltransferases in the *M. capsulatus* genome (accession NC_002977.6) and the Restriction Enzyme Database (REBASE) (39). Eleven potential MTases were predicted by REBASE (Table 2), including one that appears truncated and two that have putative frameshift mutations. Of the eight full-length *M. capsulatus* MTases (encoded by MCA0278, MCA0246, MCA1616, MCA2654, MCA2655, MCA2900, MCA3008, and MCA0550), two methyltransferases (MCA2654 and MCA2655) are in an operon, and three methyltransferases are nearby predicted REases, including one Type I (MCA0278), one Type II (MCA1616), and one Type III (MCA0550) RM systems.

**TABLE 2:** REBASE-predicted RM systems in *M. capsulatus*

Locus	Type of RM system	Annotation	Size (bp)	MTase recognition motif
MCA0269	I	Type I restriction-modification system, N-terminal domain of methyltransferase subunit M (truncated)	450	Not predicted
MCA0274	I	Type I restriction-modification system, specificity subunit HsdR	3,009	
MCA0277	I	Type I restriction-modification system, specificity subunit HsdS	1,425	
MCA0278^a^	I	Type I restriction-modification system, methyltransferase subunit HsdM	2,373	Not predicted
MCA0834	I	Type I restriction-modification system pseudogene, methyltransferase subunit HsdM (frameshift mutation)	1,457	Not predicted
MCA0836	I	Type I restriction-modification system, specificity subunit HsdS	1,251	
MCA0838	I	Type I restriction-modification system, specificity subunit HsdR	3,633	
MCA0246	II	Adenine-specific DNA methyltransferase	651	RGATCY
MCA1616^*a*^	II	DNA cytosine methyltransferase	1,038	GCGCGC
MCA1617^*a*^	II	Putative restriction enzyme	1,023	
MCA2654^*a*^^,*b*^	II	Site-specific DNA-methyltransferase	1,431	Not predicted
MCA2655	II	Site-specific DNA-methyltransferase	1,266	Not predicted
MCA2900	II	DNA-methyltransferase	915	Not predicted
MCA3008^*a*^^,*b*^	II	Putative adenine-specific methylase	3,039	Not predicted
MCA0026	III	Putative type III restriction-modification system, restriction subunit	2,742	
MCA0029	III	Putative type III restriction-modification system, methyltransferase subunit (frameshift)		Not predicted
MCA0550	III	DNA methyltransferase	4,380	Not predicted
MCA0551	III	Putative restriction enzyme	2,787	
MCA0121	IV	Conserved domain protein, putative nuclease	1,650	

^*a*^ Heterologous expression in *E. coli.*

^*b*^ Non-functional in *E. coli* based on methylome.

**Fig 3:**
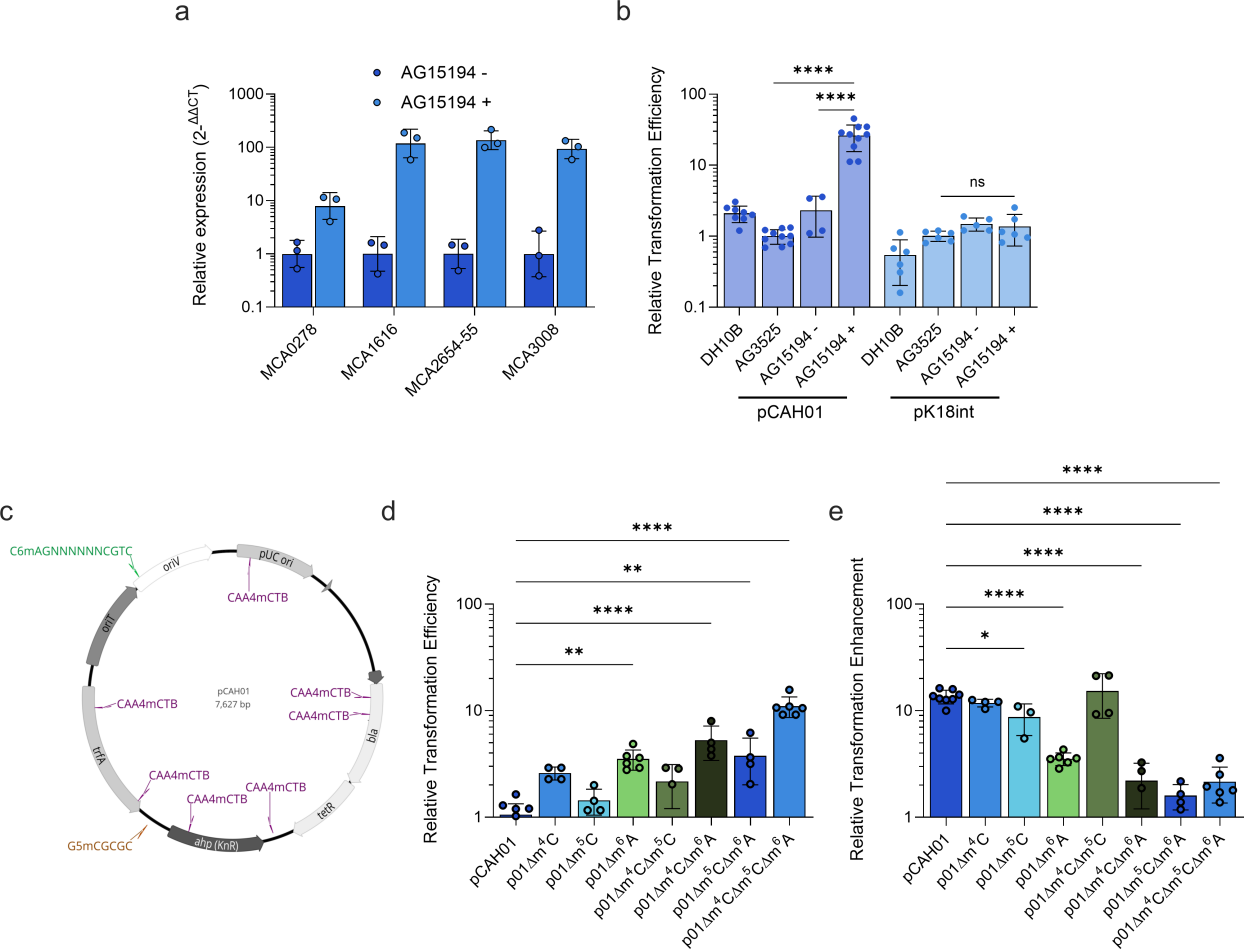
Shuttling plasmid DNA through an *E. coli* cloning strain expressing heterologous methanotroph methyltransferases improves *M. capsulatus* transformation efficiency**.** (**a**) Relative transcription of RM system methyltransferase genes in an engineered *E. coli* strain (AG15194) with (+) or without (−) induction of heterologous *M. capsulatus* methyltransferases. (**b**) Relative transformation efficiency of pCAH01 or pK18int isolated from *E. coli* cloning strains DH10B or AG15194 with (+) or without (−) methyltransferase induction. The transformation efficiency of pCAH01 from DH10B is set to 1. (**c**) *M. capsulatus* m^4^C, m^5^C, and m^6^A motifs identified in pCAH01. (**d**) Relative transformation efficiency of plasmids with mutated methylation motifs compared to pCAH01 isolated from AG15194 without methyltransferase induction. (**e**) Transformation efficiency of pCAH01 and variants with mutated methylation motifs isolated from AG15194 with methyltransferase induction relative to uninduced controls. The data represent the mean ± SEM from at least two independent experiments (*n* = 3–9). *P ≤ 0.05, **P ≤ 0.01, ****P ≤ 0.0001; ns, not significant.

Only MCA1616 had a predicted recognition sequence motif besides MCA0246 (Table 2), which is not near a predicted REase. We codon-optimized and synthesized the full-length *M. capsulatus* MTases with an arabinose-inducible promoter for iterative integration into a *dam^+^dcm^−^ E. coli* strain (AG3525) (40) using serine recombinase-assisted genome engineering (SAGE) (41). We used a *dam^+^ E. coli* strain because the Dam methyltransferase can methylate all the RGATCY motifs that are methylated in *M. capsulatus*. Four methyltransferase genes (MCA0278, MCA1616, MCA2654-55, and MCA3008) were successfully integrated into AG3525 to generate strain AG15194 (41). Methyltransferase gene transcription was increased 10–100-fold in strain AG15194 upon induction with arabinose compared to uninduced controls (Fig. 3a). Under these inducing conditions, two methylation motifs [Cm^6^AG(N)_6_CGTC/Gm^6^ACG(N)_6_CTG and Gm^5^CGCGC] were detected in the AG15194 strain that were not present in the parent AG3525 strain (Table 1), indicating that some, but not all, of the heterologous *M. capsulatus* methyltransferases were functionally expressed in *E. coli*.

To test whether strain AG15194 could improve methanotroph DNA transformation, pCAH01 was isolated from the induced AG15194 strain and transformed into *M. capsulatus* via electroporation. AG15194-derived pCAH01 showed a ~30-fold improvement in transformation efficiency compared to pCAH01 isolated from the parent AG3525 (*dam*^+^*dcm*^−^) strain or an uninduced AG15194 control (Fig. 3b), suggesting that pCAH01 is methylated in the methyltransferase-expressing strain and that methylation of the m^5^C and/or m^6^A motifs protects plasmid DNA from *M. capsulatus* RM systems. Similarly, we also observed significantly increased electroporation efficiency of the induced AG15194-derived pBBR1MCS-5 replicative broad-host-range plasmid (42) compared to uninduced controls (Fig. S1). Transformation efficiency of pCAH01 derived from *dam*^+^*dcm*^+^ DH10B had similar electroporation efficiency as the plasmid derived from the *dam*^+^*dcm*^−^ AG3525 strain (Fig. 3b); thus, the putative Type IV REase that would cut methylated DNA does not cut the 48 CCWGG sites on pCAH01 that are methylated by *E. coli* Dcm. In contrast to the replicative plasmids, the transformation efficiency of the pK18pheS suicide plasmid was slightly increased (*P* =0.0854) after being methylated in AG15194 (Fig. 3b), indicating that the efficiency of pK18pheS homologous recombination with the chromosome may be limiting suicide plasmid transformation efficiency rather than *M. capsulatus* RM systems or that restriction may be predominantly targeting sites not methylated by AG15194.

### Site-directed mutagenesis identifies the m^6^A motif as the restriction site limiting transformation efficiency

As an alternative strategy to bypass host RM systems, we iteratively removed the *M. capsulatus* methylation motifs identified in the methylome analysis from pCAH01 via site-directed mutagenesis (Fig. 3c). A DH10B-derived plasmid with a single m^5^C motif disrupted (p01∆m^5^C) did not show a statistically significant improvement in transformation efficiency compared to pCAH01 (Fig. 3d), indicating that this methylated motif, although one of the most abundant in the *M. capsulatus* genome and efficiently modified in the AG15194 strain (Table 1), does not limit transformation efficiency when unmodified. A plasmid lacking seven m^4^C sites (p01∆m^4^C) isolated from DH10B had a 2.5-fold improvement in transformation efficiency when compared to pCAH01 with the motifs intact (Fig. 3d). We also observed a significant increase in electroporation efficiency of a plasmid variant lacking the CAG(N)_6_GCTG m^6^A methylation site (p01∆m^6^A). The p01∆m^4^C∆m^6^A plasmid with both m^4^C and m^6^A mutations showed an additive increase in transformation efficiency compared to the p01∆m^4^C and p01∆m^6^A plasmids, further supporting that these two sites are likely restricted by the *M. capsulatus* RM systems if present but unmethylated. Combining the m^5^C mutation with either the m^4^C (p01∆m^4^C∆m^5^C) or m^6^A (p01∆m^5^C∆m^6^A) mutations did not improve transformation efficiency compared to the p01∆m^4^C and p01∆m^6^A plasmids, respectively (Fig. 3d). The plasmid with all motifs mutated (p01∆m^4^C∆m^5^C∆m^6^A) exhibited a 10-fold increase in transformation efficiency compared to pCAH01 (Fig. 3d), suggesting that all the unmodified motifs may be recognized by *M. capsulatus*, limiting transformation efficiency. Interestingly, when the plasmids were shuttled through the methyltransferase-expressing AG15194 strain, all plasmid variants except those with mutated m^6^A motifs had higher transformation efficiencies compared to uninduced/unmodified controls (Fig. 3e), underscoring that the pCAH01 m^6^A modification is the primary motif associated with the observed improvement in electroporation efficiency. Given that the m^4^C motifs were not modified in the AG15194 *E. coli* strain (Table 1) and removal of the methylation site improved transformation efficiency of pCAH01 derived from DH10B (Fig. 3d), we expect that additional *M. capsulatus* transformation efficiency improvements are possible via AG15194 optimization that mediates plasmid m^4^C motif modification.

### A type I RM system limits *M. capsulatus* transformation efficiency

**Fig 4:**
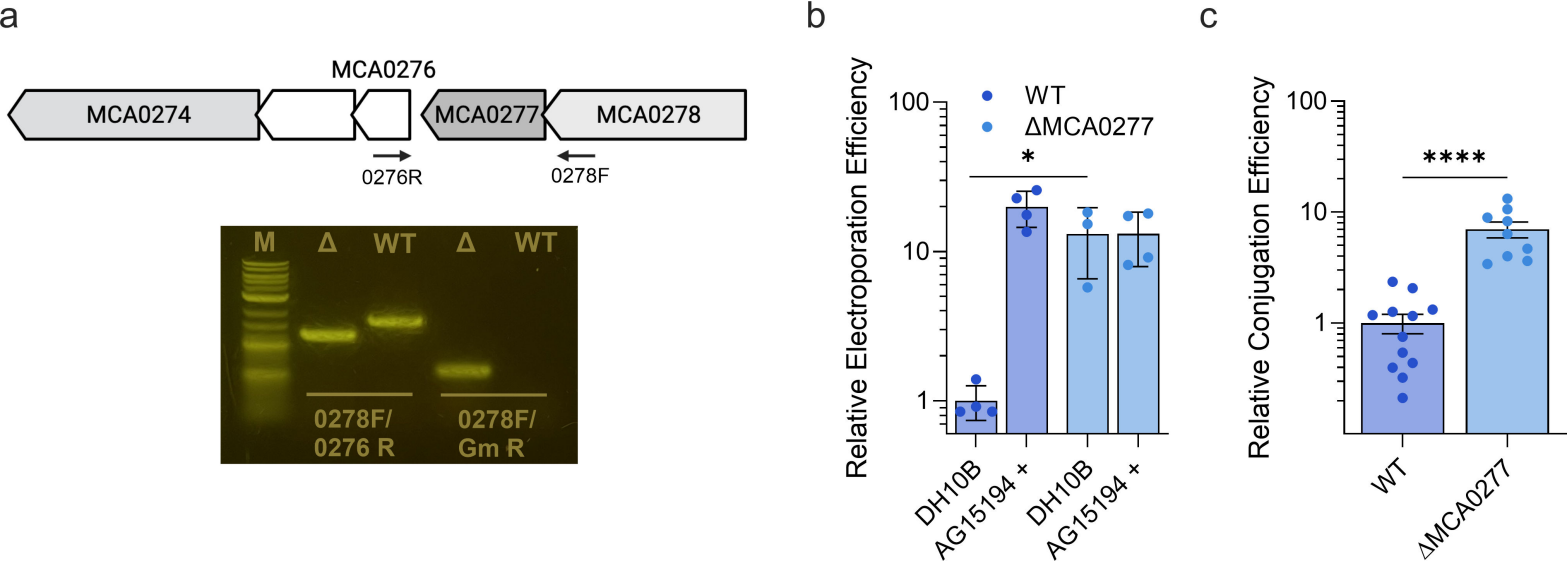
A Type I RM system endonuclease limits *M. capsulatus* transformation. (**a**) Schematic representation of the Type I RM system encoding genes associated with the m^6^A motif and PCR confirmation of a ΔMCA0277::Gm^R^ knockout strain (Δ) obtained via electroporation of a linear marker-exchange mutagenesis cassette. The location of primers (0278F and 0276R) that bind to genes used for strain confirmation are indicated in the gene schematic. We also used a reverse primer that binds within the Gm^R^ gene (Gm R) for additional strain confirmation. Wild-type (WT) *M. capsulatus* genomic DNA was used as a control PCR template for amplicon size comparison. (**b**) Relative electroporation efficiency of wild-type (WT) and ΔMCA0277::Gm^R^ *M. capsulatus* with pCAH01 isolated from AG15194 with induced methyltransferase expression (+) or plasmid isolated from DH10B *E. coli*. (**c**) Relative conjugation efficiency of WT and ΔMCA0277 calculated by comparing the transconjugant colony forming units obtained to the amount of methanotrophs mixed with S17-1 *E. coli* in the conjugation reaction. The data in panels b and c represent the mean ± SEM from at least two independent experiments (*n* = 3–12). *P ≤ 0.05, ****P ≤ 0.0001.

The Type I RM system encoded by genes MCA0278 (HsdM), MCA0277 (HsdS), and MCA0274 (HsdR) is hypothesized to be linked to the m^6^A motif identified in the *M. capsulatus* and methyltransferase-expressing AG15194 genomes (Fig. 4a). To further test the role of this Type I RM system in limiting *M. capsulatus* transformation of foreign plasmid DNA, we deleted the MCA0277 gene via electroporation of a marker-exchange mutagenesis cassette generated by PCR (Fig. 4a). Electroporation of the HsdS-deficient *M. capsulatus* ∆MCA0277 strain with pCAH01 isolated from *E. coli* DH10B showed a 10-fold improvement in efficiency relative to transformation of the same plasmid into wild-type *M. capsulatus*, comparable to the efficiencies observed in wild-type *M. capsulatus* using plasmid methylated in AG15194 (Fig. 4b). Notably, shuttling pCAH01 through AG15194 did not improve the already elevated transformation of the ∆MCA0277 strain (Fig. 4b). We also observed a sevenfold increase in pCAH01 transconjugants in the ∆MCA0277 strain compared to wild-type *M. capsulatus*, supporting that transfer of pCAH01 via conjugation is also limited by the Type I RM system (Fig. 4c). These data provide additional support that this Type I restriction endonuclease recognizes an unmodified CAG(N)_6_CGTC motif and limits *M. capsulatus* transformation. Homologs of this Type I RM system were identified in both gammaproteobacterial and alphaproteobacterial methanotroph genomes through BlastP analysis of the *M. capsulatus* MCA0274 HsdR endonuclease, including *Methylocaldum*, *Methylomonas*, *Methylomicrobium*, *Methylotuvimicrobium*, *Methylobacter*, and *Methylocystis* (Table S7).

### Bypassing native RM systems enables the generation of an *M. capsulatus* genome-wide CRISPRi library via electroporation

**Fig 5:**
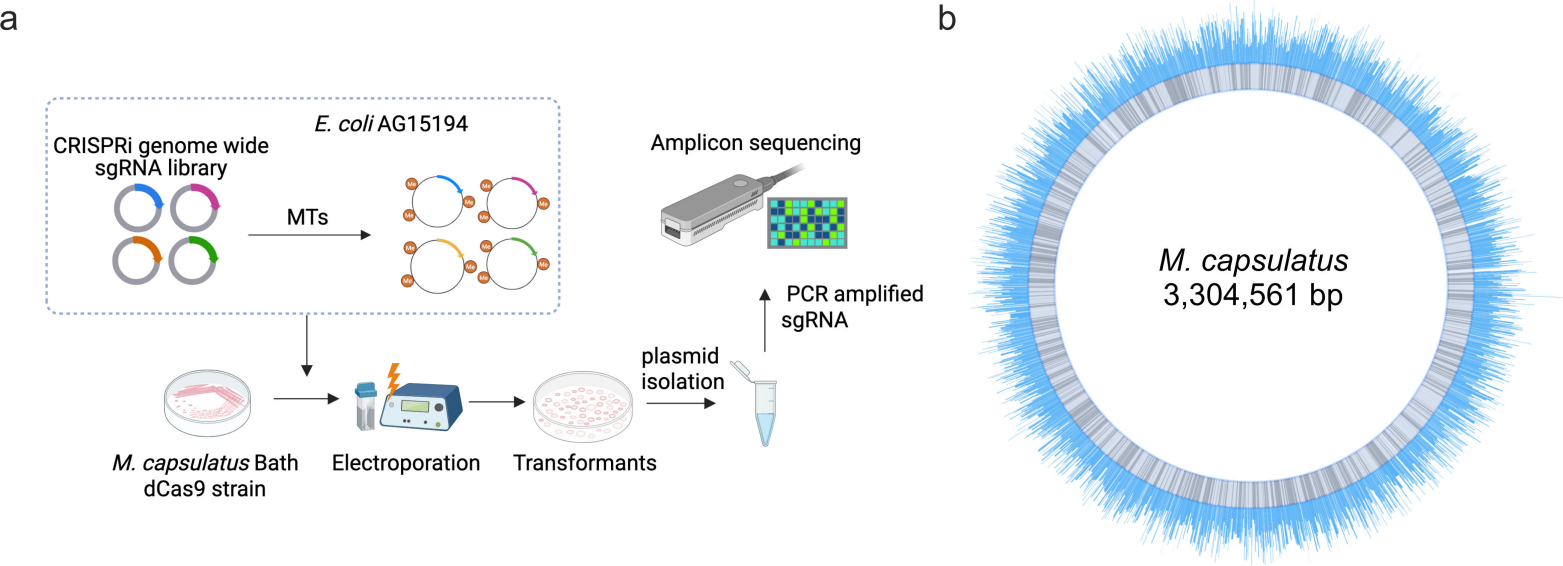
Transformation efficiency improvements enable the construction of an *M. capsulatus* genome-wide sgRNA library for CRISPR-based genomics. (**a**) Schematic diagram of the workflow for sgRNA library construction and analysis via ONT amplicon sequencing. (**b**) Circos plot of the sgRNA ONT sequence raw reads mapped to the *M. capsulatus* Bath NC_002977.6 genome. The inner ring represents the open reading frames (3,022) in the *M. capsulatus* genome, with forward and reverse orientation open reading frames highlighted in light or dark gray, respectively. The outer blue ring represents the sgRNA amplicon log-transformed raw read count mapped to the *M. capsulatus* genome.

The increased transformation efficiency (10^5^–10^6^ CFU/μg DNA) afforded by bypassing *M. capsulatus* RM systems enables high-throughput library generation (Fig. 5a). We previously constructed an *M. capsulatus* genome-wide sgRNA library in pCAH01 (p01sgRNA library) consisting of 45,798 sgRNAs; ~15 sgRNAs per *M. capsulatus* coding genes (3,022 annotated genes in the NC_002977.6 RefSeq genome) for CRISPR interference-based functional genomic screening applications (43). The p01sgRNA library was previously transferred to *M. capsulatus* via conjugation methodologies, which were labor-intensive and required several conjugation reactions to achieve complete library coverage (43). As a proof-of-concept of the utility of the transformation efficiency improvements developed here, we transferred the p01sgRNA library to the methyltransferase-expressing AG15194 strain for plasmid methylation. The AG15194-derived p01sgRNA library was then electroporated into an engineered *M. capsulatus* strain with a chromosomally integrated, inducible dCas9 gene (43). A total of 7 × 10^4^ transformants were obtained, just slightly over the amount of unique sgRNAs in the library. ONT sequencing of the sgRNAs amplified from pooled transformant gDNA identified 13,419 of the 45,798 sgRNAs (29.3% coverage) after mapping the extracted ONT sgRNA target sequences to the synthesized genome-wide sgRNA library reference (Fig. 5b; Tables S1 and S3). Mapping the sgRNA target sequences to the *M. capsulatus* genome (NC_002977.6) showed 2,987 of 3,022 open-reading frames (99.8%) had at least one respective sgRNA in the electroporation-based library (Tables S2 and S3). These data demonstrate the successful generation of an *M. capsulatus* genetic variant library via electroporation.

### Conclusions

Methanotrophs represent promising biocatalysts for GHG mitigation and conversion of CH_4_-rich gas streams. The Gammaproteobacterial methanotroph *M. capsulatus* is currently used industrially for bioconversion of natural gas to single-cell protein, and its unique ability to utilize both CH_4_ and CO_2_ makes this bacterium particularly appealing for the mitigation of the two most abundant atmospheric GHGs, CH_4_ and CO_2_. Genetic engineering of *M. capsulatus* is necessary to fully exploit the capabilities of this bacterium, but advancements in strain development have been hindered by limited genetic tools and high-throughput methodologies.

Here, we established and optimized an electroporation-based transformation method for *M. capsulatus* that enabled the transfer of replicative plasmid DNA and the integration of suicide plasmids and linear DNA cassettes into the methanotroph chromosome. This electroporation method shortens the time and simplifies the process to obtain transformants compared to conventional conjugation-based methods of DNA transfer to *M. capsulatus*.

Identification of the *M. capsulatus* methylome was used to engineer an *E. coli* cloning strain with the capacity to methylate DNA partially mirroring that of *M. capsulatus*, which significantly improved the electroporation efficiency of commonly used broad-host-range plasmids in this methanotroph. The improved electroporation efficiency was associated with methylation of the CAG(N)_6_CGTC motif. Our data suggest that if this motif is present but unmodified, an *M. capsulatus* Type I RM endonuclease limits transformation, presumably through DNA cleavage. Given that homologs of this RM system can be identified in several methanotroph genomes, its removal may be a generalizable strategy to improve methanotroph transformation.

Notably, we leveraged the advancements in *M. capsulatus* electroporation obtained in these investigations to construct a genome-wide sgRNA library for CRISPR interference-based functional genomic screening. This achievement provides proof-of-concept high-throughput genetic engineering in *M. capsulatus*, which can be mirrored in future studies with user-defined library generation and screening via negative/positive selection and/or adaptive laboratory evolution. Collectively, the tools and methodologies developed herein pave the way for efficient, high-throughput genetic and metabolic engineering of *M. capsulatus* to realize the utility of this microbial chassis in CH_4_ conversion biotechnologies.

## MATERIALS AND METHODS

### Bacterial strains and cultivation

Bacterial strains and plasmids used in this study are listed in Table S4. All *E. coli* strains were cultured in lysogeny broth (Lennox) with antibiotics (kanamycin [50 μg/mL], spectinomycin [50 μg/mL], gentamicin [10 μg/mL]), as necessary, for maintenance of plasmids and for transformant selection. *M. capsulatus* cultures were grown on nitrate mineral salts (NMS) solid medium in a stainless-steel gas chamber (Schuett-Biotech GmbH, Germany) supplied with 20% CH_4_ (vol/vol) in the gas phase and incubated at 37°C as previously described (14). Methanotroph transformants were selected on NMS solid medium containing 25 μg/mL kanamycin or 15 μg/mL gentamicin.

### Construction of pK18int for *M. capsulatus* chromosomal integration

Primers used in this study are shown in Table S5. Suicide plasmid pK18int was constructed by assembling the pK18pheS plasmid (44) backbone linearized by PCR using oCAH576/oCAH577 primers with three PCR amplicons: (i) a gentamicin-resistant gene amplified from pKD13Gm^R^ using oCAH1287/oCAH1097, (ii) a 1 kbp upstream fragment, and (iii) a 1 kbp downstream fragment homologous to an intergenic site insertion site between MCA_tRNA-Ile and MCA_tRNA-Pro amplified from *M. capsulatus* genomic DNA using primers oCAH985/oCAH1286 and oCAH1302/oCAH992, respectively. All the DNA fragments were assembled using NEBuilder HiFi DNA Assembly Master Mix (New England Biolabs). pK18int was also used as a template in PCR using primers oCAH985/oCAH992 to amplify the 3 kbp linear cassette (1 kbp upstream-Gm^R^-1 kbp downstream) for electroporation-based marker-exchange mutagenesis.

### Generation of electrocompetent cells and electroporation

*M. capsulatus* electrocompetent cells were generated following the method previously described (20) with slight alterations. Briefly, ~1/4 of a 10 μL inoculating loopful of *M. capsulatus* plate-derived biomass was densely streaked onto NMS solid medium and cultivated in a gas chamber supplied with 20% CH_4_ for 4 days at 37°C. Biomass was collected from the plate with an inoculating loop and resuspended in 20 mL ambient temperature Milli-Q H_2_O, followed by centrifugation (4,000 × *g*, 10 min at 4°C). The cell pellet was washed twice via repeated serological pipetting with 20 mL of ambient temperature Milli-Q water and then centrifuged (4,000 × *g*, 10 min at 4°C). The resulting pellet was resuspended with ice-cold Milli-Q H_2_O to OD_600_ = 200. To compare different wash buffers, the cell pellet was washed and resuspended with ice-cold 10% glycerol. For the cryogenic storage test, the Milli-Q H_2_O-washed pellet was resuspended with Milli-Q H_2_O containing 8% DMSO or 10% glycerol and stored at −80°C for 7 days. Alternatively, to assess the effect of cryoprotectants on electroporation efficiency, the water-washed cell pellet was resuspended with ice-cold 10% glycerol or 8% DMSO to an OD_600_ = 200 prior to electroporation.

Electrocompetent cells (50 μL) were mixed with plasmid or linear DNA (100–400 ng) and transferred to an ice-cold electroporation cuvette (1 mm gap, Fisher Scientific). Electroporation was performed using a MicroPulser (Bio-Rad) with a field strength set at 1.8 kV/cm and pulse duration set at 5 ms (200 ohm × 25 μF). Immediately after electroporation, 1 mL of room-temperature NMS was added to the cuvette, and the cells were transferred to a 1.5 mL centrifuge tube, followed by incubation at 37°C for 4 h in a shaking incubator. The recovered cells were spread onto selective NMS solid medium and incubated at 37°C in a gas chamber supplied with 20% CH_4_. CFUs were enumerated after 1 week of incubation. Electroporation efficiency was calculated by normalizing the total number of antibiotic-resistant transformants to input DNA or by comparing to the number of antibiotic-sensitive *M. capsulatus* cells electroporated. To assess viability after freezing and/or electroporation, cells were resuspended in NMS and serially diluted and plated on non-selective medium. Percent survival was calculated by comparing the number of CFUs after electroporation or freezing with the number of CFUs in freshly prepared electrocompetent cells before treatment.

### Biparental conjugation

Plasmids were transferred to *M. capsulatus* via conjugation with slight modifications from that previously described (16). Briefly, plate-derived biomass of *S17-1 λpir* harboring a plasmid or *M. capsulatus* was resuspended in liquid NMS mating medium (14) to an OD_600_ = 2. A volume of 500 μL of each cell suspension was mixed in a 1.5 mL centrifuge tube and incubated at 37°C with shaking (200 rpm) for 24 h. Transconjugants were enumerated via serial dilution and plating on selective NMS medium. Conjugation efficiency was calculated by comparing the total number of antibiotic-resistant transconjugants to the number of antibiotic-sensitive *M. capsulatus* cells in the conjugation cell mixture.

### *M. capsulatus* Oxford Nanopore Technologies sequencing and methylome analysis

Genomic DNA was extracted using the Quick-DNA Bacterial Miniprep Kit (Zymo Research) or Wizard HMW DNA Extraction Kit (Promega) and then prepared for Oxford Nanopore Technologies sequencing as previously described (32). The 3.3 Mbp *M. capsulatus* genome was assembled following standard workflows, and methylated motifs were identified using MIJAMP (29). The m^4^C data support the call of a CAAm^4^CTB motif. However, the m^4^C all-context model produced by ONT is the least accurate of the ONT methylation models, and other phenomena, such as GC bias and the presence of rare k-mers in *M. capsulatus,* may impact the accuracy of methylation detection. Additionally, these trivalent base ambiguities (B = C, G, and T) are uncommon in methylation motifs, and without the ambiguous base, this motif would look most like a Type III RM system, with five non-palindromic unambiguous bases. However, without additional data or updated ONT base calling models, we cannot exclude this ambiguous base.

### Construction of the *E. coli* methyltransferase-expressing shuttle strain

We utilized SAGE to generate the methyltransferase-expressing AG15194 shuttle strain (32, 40, 41). Briefly, the *M. capsulatus* genes encoding predicted DNA methyltransferases of RM systems (MCA2078, MCA2654, MCA2655, MCA1616, and MCA3008) were codon optimized, synthesized with an arabinose-inducible promoter (P*_ara_*), and cloned into suicide plasmids containing *attP* sites recognized by the Bxb1, BL3, R4, or PhiK38 recombinases, respectively, to generate plasmids pMTV2023, pMTV2025, pMTV2026, and pMTV2027 (Table S6). Plasmid pMTV2023 (P*_ara_*-MCA0278-0277) was co-transformed with pLAR074 (BXB1 recombinase-expressing, temperature-sensitive plasmid) into the *dam^+^dcm^−^ E. coli* AG3525 strain, which harbors an array of *attB* recombination sites in its chromosome (40), and recombinants were selected on LB containing kanamycin. Kanamycin-resistant recombinants were then transformed with pLAR047 for PhiC31 recombinase expression, which were selected on LB containing carbenicillin. Single carbenicillin-resistant colonies were cultured at 42°C to cure the temperature-sensitive pLAR047 and then replica-plated onto LB kanamycin, LB carbenicillin, or LB without antibiotics to screen for the loss of the kanamycin marker and pLAR047, generating a markerless strain with a methyltransferase gene recombined into the BxB1 *attB* site and ready for the next plasmid integration into the chromosomal *attB* array as previously described (40). The following three methyltransferase plasmids were iteratively integrated by co-transforming with the corresponding serine recombinase-expressing plasmid (pMTV2025+pLAR051, pMTV2026+pLAR058, pMTV2027+pLAR056), followed by kanamycin cassette removal using PhiC31 as described above, ultimately creating AG15194 (Table S6). Primers used for confirming gene integration are listed in Table S5. To assess the functionality of the heterologous methyltransferases, genomic DNA was extracted from AG3525 or arabinose-induced AG15194, and methylome analysis was performed using the ONT and MIJAMP workflows described above. Plasmids were introduced to the AG15194 methyltransferase-expressing strain via a standard *E. coli* electroporation procedure. AG15194-derived strains were grown in LB medium overnight and subcultured 1:100 in fresh LB medium with or without inducer (1 mM arabinose) in a 37°C shaker incubator for 16 h prior to isolation of methylated plasmids using a Monarch Plasmid Miniprep kit (New England Biolabs).

### Methyltransferase gene expression analysis

Quantitative reverse-transcription PCR (qRT-PCR) was used to determine methyltransferase gene expression in the AG15194 cloning strain. RNA was isolated from AG15194 cultivated in LB for 16 h after arabinose induction using the Roche High Pure RNA isolation kit following the manufacturer’s instructions (Roche). cDNA was synthesized from 1 µg RNA with the Superscript IV Vilo reverse transcriptase kit following the manufacturer’s protocol (Invitrogen). Primers used for qPCR were designed using the PrimerQuest Tool (Integrated DNA Technologies) and are listed in Table S5. A total of 10 µL qPCR reactions were prepared with Powertrack SYBR Green master mix (Applied Biosystems) following the manufacturer’s recommended template and primer input and cycling conditions in a QuantStudio 3 qPCR system (Applied Biosystems). Expression of each methyltransferase was determined by normalizing the target cycle threshold (Ct) to that of the housekeeping *rpoD* gene encoding the RNA polymerase σ^70^. Relative methyltransferase expression was calculated using the ∆∆Ct method, normalized to the lowest expressed methyltransferase gene.

### pCAH01 site-directed mutagenesis

pCAH01 was amplified by PCR using oCAH1840/oCAH1841 and oCAH1842/oCAH1843 to generate two fragments, which were assembled using isothermal assembly to remove three CAAm^4^CTB sites. Four additional CAAm^4^CTB sites were sequentially mutated using a Q5 Site-Directed Mutagenesis Kit (New England Biolabs) and primers oCAH1880 to oCAH1887, generating p01∆m^4^C. A single Gm^5^CGCGC site was then mutated in pCAH01 and p01∆m^4^C using primers oCAH1846/oCAH1847 to form p01∆m^5^C and p01∆m^4^C∆m^5^C, respectively. The Cm^6^AG(N)_6_CGTC site in pCAH01, p01∆m^4^C, and p01∆m^4^C∆m^5^C was then mutated using primers oCAH1957/oCAH1958 to form p01∆m^4^C∆m^6^A, and p01∆m^4^C∆m^5^C∆m^6^A, respectively.

### Generation of the *M. capsulatus* ∆MCA0277 knock-out strain

The 1 kbp upstream and downstream regions of the MCA0277 gene in *M. capsulatus* chromosome were amplified from *M. capsulatus* genomic DNA using primers oCAH1959/oCAH1960 and oCAH1963/oCAH1964, respectively. MCA0277 is in an operon with the upstream methyltransferase (MCA0278) with an overlapping stop/start codon. Thus, the adenine of the start codon of MCA0277 was maintained to avoid disruption of MCA0278 translation. The gentamicin-resistant gene was amplified from pKD13Gm^R^ using oCAH1961/oCAH1962. An isothermal assembly of the three DNA fragments was used as a template to amplify a 3 kbp cassette (1 kbp upstream-Gm^R^-1 kbp downstream) for marker-exchange mutagenesis using oCAH1959/oCAH1964. The DNA fragment was purified using the Monarch PCR&DNA clean-up kit (New England Biolabs), and 400 ng was electroporated into *M. capsulatus* as described above. Gentamicin-resistant transformants were screened with primers oCAH1965/oCAH1966, followed by Sanger sequencing to confirm replacement of the MCA0277 locus with the gentamicin cassette.

### CRISPRi library generation via electroporation

The previously constructed CRISPRi library (43) was isolated from *E. coli* DH10B and transferred to the electrocompetent AG15194 strain. Prior to plating on selective medium, the recovered electroporated cells were also serially diluted to determine the number of transformants/library coverage. A lawn of transformants (2.68 × 10^10^ transformants, five orders of magnitude greater than the synthesized sgRNA library) was resuspended in LB to OD_600_ = 30 and subcultured 1:100 in fresh LB containing 1 mM arabinose for 16 h to methylate plasmid DNA. The methylated p01sgRNA library was then isolated from 5 mL of induced AG15194 culture using the Monarch Plasmid Miniprep kit (New England Biolabs). The p01sgRNA library was electroporated into the *M. capsulatus* strain with an integrated P*_tet_*-dCas9 (43) to generate the methanotroph CRISPRi library. *M. capsulatus* transformants were quantified via serial dilution plating and pooled for genomic DNA extraction and long-term −80°C storage. Library composition was determined via ONT amplicon sequencing and bioinformatic analyses as described previously (43). In total, 118,829 extracted sgRNA target sequences were aligned to either a custom reference library consisting of the 45,798 sgRNA sequences or the *M. capsulatus* Bath genome (NC_002977.6) to determine sgRNA library coverage.

### Illustrations

Data was graphed using GraphPad Prism 10 software. Workflow schematics were created with BioRender.com. Circos plots were generated using Circa (https://circa.omgenomics.com).

### Statistical analysis

Statistical analysis was performed using GraphPad Prism 10 software. The data were analyzed using unpaired t tests (two groups) or one-way ANOVA, followed by post hoc Tukey HSD test (≥three groups). Data were considered statistically significant when *P* ≤ 0.05.

## Data Availability

All data are available in the main text or the supplemental material. The *M. capsulatus* Bath and AG15194 ONT raw genome sequencing data pod5 files can be downloaded from Zenodo at https://doi.org/10.5281/zenodo.20315400.

## SUPPLEMENTAL MATERIAL

Supplemental material aem.00538-26-s0001.docx Figure S1; Tables S3 to S6.

Tables S1, S2, and S7 aem.00538-26-s0002.xlsx Mapped sgRNA reads and BlastP results.

ASM does not own the copyrights to Supplemental Material that may be linked to, or accessed through, an article. The authors have granted ASM a non-exclusive, world-wide license to publish the Supplemental Material files. Please contact the corresponding author directly for reuse.
