# Supplementary material for: Evading native restriction-modification systems improves electroporation efficiency of the methanotroph Methylococcus capsulatus Bath
Source: Appl Environ Microbiol. 2026 Jun 12;92(7):e00538-26. doi: 10.1128/aem.00538-26 (PMC13390346; doi:10.1128/aem.00538-26)
Supplement: Supplemental material — Figure S1; Tables S3 to S6. [file aem.00538-26-s0001.docx]

**Yu et al. Supplemental Material**

**Evading native restriction-modification systems improves electroporation efficiency of the methanotroph, *Methylococcus capsulatus* Bath.**


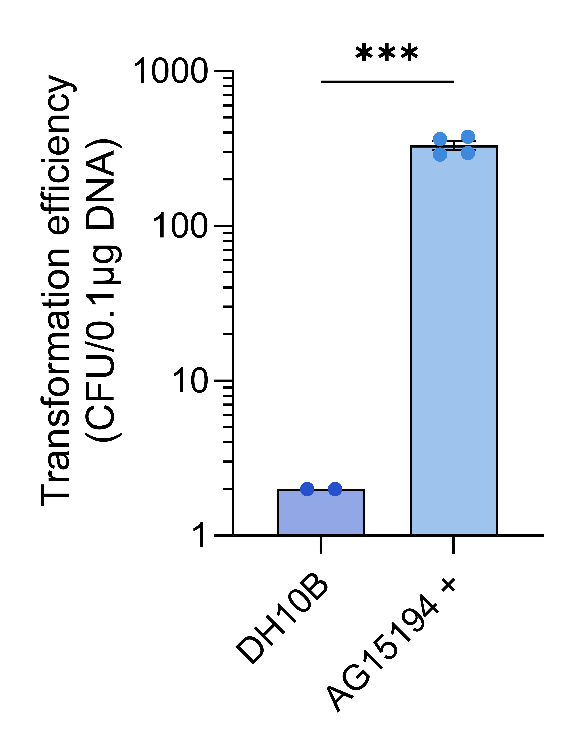


**Figure S1. Enhanced transformation of methylated pBBR1MCS-5 into M. capsulatus.** Relative transformation efficiency of pBBR1MCS-5 plasmid isolated from AG15194 with induced methyltransferase expression (+) or plasmid isolated from DH10B E. coli. The data represents the mean ± SEM from two independent experiments (n=4). ✱✱✱p ≤ 0.001.

**Tables S1 and S2. sgRNA target sequence mapping read counts.** Excel file

**Table S3. Electroporation-based genome-wide sgRNA library parameters.**

| Synthesized sgRNA library size | 45,798 |
| --- | --- |
| Total number of *M. capsulatus* transformants | 70,000 |
| Total ONT sgRNA sequence reads (Q > 12) | 425,506 |
| Total ONT sgRNA sequence reads after filtering | 351,891 |
| Total extracted sgRNA target sequences | 351,822 |
| Extracted sgRNA target sequences mapped to synthetic sgRNA library | 235,104 |
| Unique extracted sgRNA target sequences mapped to synthetic sgRNA library | 13,419 |
| sgRNA library coverage | 29.3% |
| *M. capsulatus* coding DNA sequences | 3,022 |
| Genes with at least 1 mapped extracted sgRNA target sequence | 2,987 |
| Genome coverage | 99.8% |

**Table S4. Strains and plasmids.**

| Name | Description | Reference |
| --- | --- | --- |
| Strains |  |  |
| *Methylococcus capsulatus* str. Bath | Wild type | Lab stock |
| *∆MCA0277* | M. capsulatus Bath with knock-out MCA0277 | This study |
| *E. coli* str. DH10β | F- *mcrA* Δ(*mrr-hsdRMS-mcrBC*) Φ80*lacZΔM15* Δ*lacX74* *recA1 endA1* *araD139* Δ(*ara leu*) 7697 *galU galK rpsL* *nupG* λ- | Zymo Research |
| AG3525 | *E. coli* WM3188  (DH10B transduced to *leu*^+^ *araC*^+^) Δ*dcm::frt poly-attB* | (Riley et al. 2023) |
| AG15194 | AG3525 with integrated methyltransferases from pMTV2023, pMTV2025, pMTV2026, and pMTV2027 | This study |
| Plasmids |  |  |
| pCAH01 | *P_tetA_ bla tetR* CoE1*ori* F1 *oriV oriT trfA ahp* | (Henard et al. 2016) |
| pBBR1MCS-5 | Mobilizable broadh-host-range shuttle and expression vector, Gm^R^ | (Kovach et al., 1995) |
| pK18mobpheS | Marker exchange mutagenesis suicide plasmid with *pheS* counterselectable marker | (Lee et al. 2024) |
| pK18::int | pK18 suicide plasmid with integration cassette for *M. capsulatus* Bath genome insertion | This study |
| pLAR047 | Ap^R^; Temperature sensitive; P_ENO_-φC31 recombinase | (Riley et al. 2023) |
| pLAR051 | Ap^R^; Temperature sensitive; P_ENO_-BL3 recombinase | (Riley et al. 2023) |
| pLAR056 | Ap^R^; Temperature sensitive; P_ENO_-φK38 recombinase | (Riley et al. 2023) |
| pLAR058 | Ap^R^; Temperature sensitive; P_ENO_-R4 recombinase | (Riley et al. 2023) |
| pLAR074 | Ap^R^; Temperature sensitive; P_ENO_-BXB1 recombinase | (Riley et al. 2023) |
| pMTV2023 | Km^R^; oriR6K non-replicating integration plasmid with P*_ara_*-MCA0278-0277-BXB1*attp* | This study |
| pMTV2025 | Km^R^; oriR6K non-replicating integration plasmid with P*_ara_*-MCA2654-2655-BL3 *attp* | This study |
| pMTV2026 | Km^R^; oriR6K non-replicating integration plasmid with P*_ara_*-MCA1616-R4 *attP* | This study |
| pMTV2027 | Km^R^; oriR6K non-replicating integration plasmid with P*_ara_*-MCA3008-φK38 *attP* | This study |
| p01∆m^4^C | pCAH01 with mutated m^4^C sites | This study |
| p01∆m^5^C | pCAH01 with mutated m^5^C site | This study |
| p01∆m^6^A | pCAH01 with mutated m^6^A site | This study |
| p01∆m^4^C∆m^5^C | pCAH01 with mutated m^4^C and m^5^C sites | This study |
| p01∆m^4^C∆m^6^A | pCAH01 with mutated m^4^C and m^6^A sites | This study |
| p01∆m^5^C∆m^6^A | pCAH01 with mutated m^5^C and m^6^A sites | This study |
| p01∆m^4^C∆m^5^C∆m^6^A | pCAH01 with mutated m^4^C, m^5^C and m^6^A sites | This study |

**Table S5. Primers^a^.**

| Primer name | Sequence |
| --- | --- |
| oCAH447 int Fc | GACGACGACCGTCACAAA |
| oCAH448 int Rc | GTTTTCGTGATTCGCCGC |
| oCAH985 intUP F | acagctatgacatgattacgaattcGCGCACGATCCGTATTCC |
| oCAH1286 intUP R_Gm^R^ | gatccccaattcgATCTGTCGAAGCGTGCCG |
| oCAH1287 Gm^R^ F_IntUP | gcttcgacagatCGAATTGGGGATCTTGAAG |
| oCAH1097 Gm^R^ R_intDwn | ttgggacaagagccaagggccttgcCGAATTAGCTTCAAAAGC |
| oCAH1302 intDWN F_Gm^R^ | gcgcttttgaagctaattcgGCAAGGCCCTTGGCTCTTG |
| oCAH992 intDWN R | taaaacgacggccagtgccaagcttATCTCTCGCAAGACGGCG |
| oCAH576 pK18mobsacB F | AAGCTTGGCACTGGCCGT |
| oCAH577 pK18mobsacB R | GAATTCGTAATCATGTCATAGCTGTTTCCTG |
| oCAH1840 pUCori_Kn^R^ F | cgacaccatcgaatggccagatgaCGTTCCACTGAGCGTCAGACC |
| oCAH1841 trfA_SDM R | ACAGCGTGCAAtTGGCTCCC |
| oCAH1842 trfA_SDM F | GGGAGCCAaTTGCACGCTGT |
| oCAH1843 KnR_pUCori R | TCATCTGGCCATTCGATGGTGTCG |
| oCAH1846 5mC_SDM F | AGTTCGTAGAatatatCGTGCGTCCCG |
| oCAH1847 5mC_SDM R | GCCGATAAACAGAGGATTAAAATTG |
| oCAH1880 4mC_SDM_pUCori F | CAAGAGCTACcaattcTTTTTCCGAA |
| oCAH1881 4mC_SDM_pUCori R | ATCCGGCAAACAAACC |
| oCAH1882 4mC_SDM_Kn^R^ DWN F | TCTGATCCTTcaattcAGCAAAAGTTC |
| oCAH1883 4mC_SDM_Kn^R^ DWN R | TCACGCATCTTCCCG |
| oCAH1884 4mC_SDM_Kn^R^ F | GTTTCAGAAAcaattcTGGCGCATCG |
| oCAH1885 4mC_SDM_Kn^R^ R | ATGGCAAAGGTAGCGTTG |
| oCAH1886 4mC_SDM_trfA F | CGCTGCCTCGcaattcTTCGCAGGCT |
| oCAH1887 4mC_SDM_trfA R | GCCTGGTGGAACACGCCTG |
| oCAH1929 Gm^R^  R | GTAACATGCGAAACGATCCTC |
| oCAH1957 OriV_6mA SDM F | cgccgccGAAGCCGTGTGCGAGACAC |
| oCAH1958 OriV_6mA SDM R | tttccgGCGCGTTTGCAGGGCCAT |
| oCAH1959 MCA0277 UP F_pK18 | acagctatgacatgattacgaattcGTTCCAGCCGCTGCGCCG |
| oCAH1960 MCA0277 UP R_pKD13 | agctccagcctacacTCACGCGCCCCCCACGAT |
| oCAH1961 pKD13 F_MCA0277 UP | atcgtggggggcgcgtgaGTGTAGGCTGGAGCTGCTTC |
| oCAH1962 pKD13 R_MCA0277 DWN | gcgcagccgaaccctgctATTCCGGGGATCCGTCGAC |
| oCAH1963 MCA0277 DWN F_pKD13 | acggatccccggaatAGCAGGGTTCGGCTGCGC |
| oCAH1964 MCA0277 DWN R_pK18 | taaaacgacggccagtgccaagcttCACGGTCGTACTTGAGGGC |
| oCAH1965 MCA0277 F | GCCTGTTGCAGAAGATCG |
| oCAH1966 MCA0277 R | AGTCTATCTCCCGGCCAC |
| oCAH2001 MCA0278 CO_RT F | ATCCTTCCTTTCACAGTCCTTC |
| oCAH2002 MCA0278 CO_RT R | CTGCTCAGCGACGTTATTCT |
| oCAH2003 MCA2654 CO_RT F | GTGGAGTCTTTGGGCATTTAC |
| oCAH2004 MCA2654 CO_RT R | CATGCGTCCCAATATGGAATC |
| oCAH2005 MCA2655 CO_RT F | AATCTTGGCGATGGGTTTC |
| oCAH2006 MCA2655 CO_RT R | GATGACATGGCAACGTAGATAG |
| oCAH2007 MCA1616 CO_RT F | GAACGTGAAGGGACTGTTAATG |
| oCAH2008 MCA1616 CO_RT R | CAACTGGTGCGTAACTGAATAG |
| oCAH2009 MCA3008 CO_RT F | CTAGCAGTATCGTCTTGGTTTG |
| oCAH2010 MCA3008 CO_RT R | CGATAGCCTCTGGAAGAGTAG |
| oCAH2059 E coli_rpoD RT F | AACCGTCGTATGTCCATCGG |
| oCAH2060 E coli_rpoD RT R | AGACCGATGTTGCCTTCCTG |
| oMTV2811 MCA0278-0277 F | CACTGCGTGCTGAGATTTGG |
| oMTV2812 MCA0278-0277 R | GGTGAAACAAGCCCAGACTG |
| oMTV2815 MCA2654-2655 F | ACGAGGTGATTCCATATTGGGAC |
| oMTV2816 MCA2654-2655 R | GTTGGGTCGGAGTTAAGTGGTC |
| oMTV2817 MCA1616 F | GGTATGGACCTTGGTTTCCGTG |
| oMTV2818 MCA1616 R | CTCGTCTTCATCTAACGCAGCA |
| oMTV2819 MCA3008 F | CCCTCGACTTTGCATCTGTG |
| oMTV2820 MCA3008 R | CAACGGAACCTCTACATGCGA |

^a^*Lowercase sequence are homology arms for isothermal assembly.*

**Table S6. Synthetic, codon-optimized methyltransferase gene sequences.**

| MCA0278-0277 | ATGAAAAGTGGGAAGGTCCAGGATCAGAGTCAGATCAAATGGATTTCTGATTTTATTTGGAATATTGCAGACAACCGCCTTCGTGACGTCTATGTACGTGGTAAATACCGCGACGTTATCCTTCCTTTCACAGTCCTTCGTCGCTTGGACGCTGTACTTGAAGAGACTAAACAGAAGGTCTTGGAGCGCAAGCGCTTCTTAGACAAGAATAACGTCGCTGAGCAGGACGGAGCTCTGCGCATGGCAGCTGGGCAAGCCTTTTACAACGTATCGGAATTCACACTGGCGAAACTGAAGGCTTCTAGTCAGGGCCAACGCCTGCGCGAAGATTTCATCGCTTACCTTGACGGATTTAGTCCAAATGTACAAGAAATTCTTACAAAATTCAAGTTTCGTGATCAGATTCAGACGCTTGTTGACGCCCATGTTCTTGGGTACTTGATCGAAGATTTTCTTGACCCGGAAATCAACCTGTCTCCCCTGCCCGTTAAAGATGCCGATGGACGTATCAAGCTTCCCGCACTGGATAACCACGGTATGGGTACCGTGTTCGAGGAACTTATCCGTCGCTTTAACGAAGAGAATAATGAGGAAGCTGGTGAACACTTTACGCCACGTGATGTAGTGCGTTTGATGGCAAAATTAATGTTCATGCCAGTCGCAGACCAAATTCAATCGGGAACCTACTTACTTTACGACGGCGCATGCGGGACTGGCGGGATGCTGACAGTAGCTGAGGAGACACTGCGTGAGCTTGCTGAAGAGCAAGGGAAAGAGGTTTCCATCCACTTGTTTGGACAAGAGATCAACCCGGAAACTTACGCCATTTGTAAAGCGGACTTGTTGCTGAAAGGAGAGGGCGATGAAGCTGAGCATATCGTAGGGGGTGCCGATAAGTCGACCCTTTCAAACGACCAATTTCGCAGCCGTGAGTTCGATTTCATGATTTCCAATCCCCCGTACGGCAAATCTTGGAAGACCGACTTGGACCGTATGGGTGGGAAGAAGGGTTTCAACGATCCCCGTTTTATCGTGTCCCATTCCGGGGACCCTGAGTTTAAGTTGATCACGCGTTCTTCCGATGGCCAGCTGATGTTTTTAGTGAACAAACTTCAAAAAATGAAACAACACTCTCCATTGGGTTCGCGTATCGCCATTGTCCACAACGGGTCGGCACTGTTCACAGGAGACGCCGGCCAAGGTGAATCTAATATCCGTCGCTGGATTCTTGAAAATGACTGGTGTGAAGCCATTATCGCCTTACCCTTGAACATCTTTTACAATACAGGGATTGCCACTTACATTTGGGTCCTGACCAATCGTAAAGCCAAACACCGTAAGGGGCGTGTACAGCTGATCGACGCCACTCGTTGGTTTCAACCACTTCGCCGCAATTTGGGCAAAAAAAATTGTGAATTATCCGAGGCGGATATTCAACGCATTCTTGACCTGTACCTGGGGCAACCTCAAAACACCCCGGAATGCAAGTGGTTTGACAATGCCGACTTCGGCTATTGGAAGATCACCGTTGAACGCCCTTTGCGCCTTAAGTCACAATTGACACGCCGTGCAATTGAAACCCTGCGTTTCGCCTCGGGAGATGAGGCACTGCGTGCTGAGATTTGGGCAAAATATGGCGACAAACTTTATGCGGAATTCTCCAAACTGAAGCCGGAAATCGAGGCCTGGTTAAAGGGCGACACGGGGGAGGACGATGACGAGCCTGAGGGAGACGAAGACGAAGGGGCCCCTGCAAAAAAGGCTGTACCAGAGAAGCGTCGTAAGAAATTACTGGATGTTTCTACGTGGCAACGTGACAAGACTTTGATCGAGTTAGCTCTGTTAGCTCAGCAAGAGCTTGGTGACGGTGTGTTTGACGACCACAACGATTTTCGCGCCCGTTTTGAAGCCGCCATGGCAAAACATGGCAAAAAGTTAGCGGCCGCCGAGAAAAAGGCCATTTTCAAAGCGGTTTCCTGGCGCGACGAGACGGCCCCTCCCGTAATCGCCAAGCGTACCAAATTGAAAAAGGACGAACCATTCGAACCAGGCCTGGATGGCGTGTATCTGGAGGTAGCAGGTAAAGACCGTTTCCTGGTCGAATATGAGCCCGATGCCGACCTGCGTGACACGGAACAGGTGCCACTGAAAGAACCGGGGGGCATCGACGCTTTTTTCCGTCGTGAGGTACTTCCCCACGCACCTGATGCATGGATCGCTCGTGATAAAACTCAAATCGGGTATGAGATTTCATTTGCACGCTATTTCTATAAGCCTGCACCTCTTCGCACATTGGACGAAATTCGTGCGGACATTTTACGCCTTGAACAACAGACCGAGGGGTTGCTTCAGAAAATCGTGGGTGGCGCTTGA    ATGGCAGTCGAATCGACTTACCCTAATTATCAACCAACGCGTAGTCGCTGGGTCCCACGCGTACCTGAGCATTGGAGCCTGTTGCGTGCGAAAAATTTTTTGCGCGAGATTGATGATCGTTCGAAAACGGGTGAGGAGACTTTACTGTCGATGCGTATGCAACGTGGTCTGGTCCCACATAACGACGTCTCAGTGAAGCGCATCGCACCGGAGAATTTGATTGGCTACAAGAAAGTGCAACCAAATGAGTTAGTTTTGAATCGTATGCAAGCCGGGAATGCTATGTTTTTTCGCAGCCGTCAGTCTGGGCTTGTTTCACCCGATTACGCGGTCTTCCGCTTGTTACGTGATGATAACCCAGAATACCTTGGACACCTTTTCCGTTCTTGGCCGATGCGCGGTTTATTCCGTTCTGAGTCCAAGGGACTTGGCACGGGTACTAGCGGATTCCTGCGCCTTTATAGTGACCGTTTTGCCTCATTAGAAATTCCCCTTCCCCCACGCCCGGAACAAGATCAGATTGTGGCTTACCTTCGCGCCCAGGACGCGCATATCGCTCGTTACATCTTGGCGAAGCGCGAATTAATTAAACTTCTGACTGAGCAGAAGCTGACCATCATCGACCATGCTGTGACCCGTGGCCTGGACCCGAACGTTCGCCTGAAGCCTAGTGGTATCCAATGGTTGGGGGAAGTCCCCGAGCACTGGGAGGTGGCGTCTATTAAGCACATCGCCGATGTACGCTTTTCCGGCGTTGACAAGCATTCCAACGATGACGAAACACCCGTGCGCTTATGCAACTATACTGATGTCTATAAGAATGAACGCATTACTGCGGATATGGATTTAATGCGTGCGACCGCTACCGCAGCTGAGATTGCACGTCTGACATTAAAAGCTGGGGATGTAATTTTAACAAAAGATTCCGAAACACCTGATGATATTGGTGTTCCTGCCTGGGTTCCTGAAGACCTGCCCGGGGTGGTCTGTGCGTACCATCTGGGATTATTGCGCCCTGTACCCCAGCGTGTATTGGGAGAATTCTTATTTCGTAGTATTGGGAGTACGCGCACAGCCCAGCAGTTTCATGTATTAGCCACCGGCGTAACACGTTTTGCTTTGGGAAAACACGATGTTAAGAATGCTATCATTGCCTTGCCACCTGTTGAAGAACAACAAGCTATCTGTCGTTGGATCGTGGAAGAATGCCAACCATTAGACGAAGCCATTGCACGCGCAGAGGAGGAAATTCAGTTGATCCGTGAGTACCGTGACCGCTTGATCGCAGACGTCGTAACAGGCCAGATTGACGTGCGTGGCTGGCGCCCCGGCCCAGATGATGTAGTGAGTGATGAAGAACTTGCCGCATTGGGAGACGATGAGGCAGACTTGGGAGAGGATGAACCAGGAGATGATGGAGAGTGA |
| --- | --- |
| MCA2654-2655 | ATGCATAGTGAGACGCAAGTAGCGAAGATTGCGGCTTCTATTGTCGAGTACGGTTGGACCTCGCCTATCCTTGTCGACGGGGATAACGGTATTATTGCGGGACATGGGCGTCTGGCGGCTGCGCGTAAGTTGGGGTTGGCCGAGGTTCCAGTGATCGAACTGGCACATTTAACTCCCACGCAAAAACGTGCCCTTGTATTGGCTGACAATCGTTTAGCCTTAGAGGCCGGGTGGGATGAAGAGTTACTGGCACTGGAACTTGCTGAACTTAGTGATGCGGGATATGACCTTGCGCTTACAGGGTTTGACGACGGGGAGATTGAGGCGTTTTTATCGGAGCCCCAACCTCATGAAGATACGTCCGCAGAGGCTGATGCAGGCAAGGATGATACTACTGATGATGTGCCTGACGCCCCAGTGGTACCCGTAAGCCGCCCTGGAGATGTATGGTGCTTAGGATCACATCGCTTAATCTGCGGTGACGCAACAGATCCTGCAGTAGTTGCTGCACTTATGCGTGGCGAGCGTGCTAAGTTATGTTTCACCTCCCCTCCATATGGCAATCAGCGTGACTACACCACGGGAGGTATTGCTGACTGGGATGCCTTAATGCGTGGAGTCTTTGGGCATTTACCCATGGCAGAAGACGGACAGGTTCTGGTCAATTTAGGCTTGATCCACCGCGACAACGAGGTGATTCCATATTGGGACGCATGGCTGGGATGGATGCGTGCCCAGGGATGGCGTCGCTTCGCTTGGTACGTGTGGGACCAGGGACCCGGGATGCCCGGAGATTGGGCAGGACGCTTCGCTCCGTCTTTTGAGTTTGTCTTTCACTTCAACCGTCAGAGTCGTAAGCCCAACAAGATCGTCCCATGCAAGCATGCGGGACAAGACTCGCATCTGCGTGCGGATGGAAGCTCCACCGCTATGCGCAACAAAGACGGTGAAGTTGGCGGTTGGACCCACGCAGGCTTACCGACCCAGGATACGCGCATCCCAGATTCCGTCATTCGCGTGATGCGTCATAAGGGGAAAATTGGGAAGGGGATCGATCACCCGGCAGTGTTTCCTGTAGCTTTGCCGCAATTCGTCATGGAAGCTTACTCCGACGAAGGCGCGGTCGTCTTCGAACCATTCGGCGGCTCGGGCACTACGATGTTGGCTGCGGAGCGCACGGGTCGCGTCTGCCGTAGCGTGGAGATTGCGCCCGAATACGTGGACGTTGCGATTAAGCGTTTTCAACAAAATCACCCCGATGTGGCTGTGACTCTGTTGGCTAGTGGACAGAGTTTTGCCGAAGTAGCTGCTGAACGCGAGCGCGAAGCGGAGGTAGTAGCCTGA    ATGACAAATAGCTGGTTTGCCGACAAGATCGAGCAGTGGCCTACGGCTAAACTTGTTCCGTACGCACGTAATGCGCGCACTCATTCTGACGAGCAAATTGCACAGATCGCAGCTTCTATTGCTGAGTTTGGCTTCACAAATCCCATCCTGGCCGGGAGCGACGGAGTCATTGTGGCTGGACACGGCCGTCTTGCAGCGGCGCAAAAATTGGGTTTAGATATTGTTCCCGTTGTCGTACTGGACCACTTAACTCCGACCCAACGCCGCGCATTAGTTATTGCGGATAATCGTATTGCCGAGAATGCTGGATGGGACGATGCACTTTTGCGTGTGGAGTTAAAGGCGCTTCAAGATGAGGGATTTGACTTGGACTTAACAGGTTTTGACGCAGGGGCGCTGGCCGATCTTCTGGCCGGAGATGAACCCGCTAACGAAGGTCAGACGGACGACGATGTCGTCCCAGAAGTAAGCGAGACTCCGGTCTCACGCCCGGGAGATGTCTGGTTATTAGGACCTCACCGTCTGTTGTGCGGTGATGCTACTGTGGCCGATAGTTATGCACGTCTGTTAGCAGGTGACCCAGTTGATATGGTATTCACGGACCCGCCCTACAACGTCAACTACGCCAACTCCGCAAAAGACAAAATGCGCGGCAAAGATCGTGCGATCCTTAATGATAATCTTGGCGATGGGTTTCACGACTTTTTACTGGCTGCGCTTACCCCAACCGTCGCCAATTGTCGTGGCGCTATCTACGTTGCCATGTCATCGTCCGAGCTTGATACCTTGCAGGCCGCATTTCGCGCAGCTGGTGGTCACTGGAGCACATTTATTATTTGGGCCAAGAATACGTTTACGCTTGGTCGCGCTGATTATCAACGCCAATATGAGCCAATTCTTTACGGTTGGCCGGAAGGAGCACAACGCCACTGGTGCGGTGATCGCGATCAGGGCGATGTGTGGCAAATTAAGAAGCCGCAGCGTAATGATCTTCACCCCACTATGAAGCCAGTCGAGTTAGTCGAACGCGCGATCCGCAATTCCTCCCGTCCAGGGGCCGTGGTGCTGGATCCTTTTGGTGGTAGCGGGACCACCTTGATTGCAGCGGAAAAGGCTGGCCGTGTCGCCCGCCTGATTGAGCTGGATCCCAAATATGTGGATGTAATCGTCCGTCGCTGGCAAGACTGGACGGGCAAACAAGCTACCCGTGAAGCGGACGGATTGGCCTTCGACCAAGCGGCAACCAGCTCGGCAACAATCTTGTAG |
| MCA1616 | ATGGCTATTGAGCACCACATCACTCTGGAAACACGCGCAGATACCTTGGCATACGCGTCGATTAAAGCCGCCTCTTCGTTAACGATCTCCAAAGCCAAGGCGTCCGCAAAGAAAGCCACCGCGTTCAGCGTGATCAGTTTGTTTTCGGGCTGTGGCGGTATGGACCTTGGTTTCCGTGGTGGCTTTGAGTTTCTGGGAAAGCGCTATGCAAAATTACCATTTAATGTCATTTGGGCTAACGAGATTAACGAGGCCGCGTGTCAAACCTATCGCCGTAACCTGGGGTCTCACATCCACCATGGGGATATCTGGCAGATGATGGATTCCTTACCACCCGAGGCAGACGTAGTGATTGGGGGGTTCCCCTGCCAGGACATTTCGGTTAACGGCAAGGGTGCCGGAATTAATGGACAACGTTCGGGTTTATACCGTGCAATGGTGGAGGTTGTGCGTCGTGTGCGCCCTAAGGTATTCGTGGCTGAGAACGTGAAGGGACTGTTAATGCGCCATCATGCAGACGCATTAAAAACCGTCTTGGATGACTTCTCGGCGTTGGGCTATTCAGTTACGCACCAGTTGTACTTAGCCGCCGATTATGGTGTCCCCCAGACCCGTGAGCGCGTTTTGATTGTAGGTACATTGCCCGGTGTAAAACCGTTTGAGCCGCCCAAGCCTATTATTTCCCCTTCGCGTTATATTACGGCTAAACAGGCGATTGACGACCTTGCTGCGTTAGATGAAGACGAGGAGATTAACCACGTATGGAGTCGCGCCGGAAAGTCTCCTGAACAGGGTAATCGCCGCTTGATCGCTGACCGTCCTGGTTACACCATCCGTGCGGAGTGCCATGGTAATATCCAGTGGCACTATGAACTGCCTCGCCGTATCAGTATGCGTGAGGCCGCTCGCATCCAGTCATTTCCTGATGAGTTTATTTTCGCCTCTAAGCTGCGTGAGACGGAGCGTCAAATTGGGAATGCCGTTCCACCAGTGTTGGCGTGGCACATTGCTAAAGCGGTTGCACAATGTCTGAAGTGA |
| MCA3008 | ATGACCACTGTGAAAGCACCAAAAAAACTTATTGAGGTCGCGTTACCTTTGGACGCAATCAATGAAGCTAGCGCGCGCGAGAAAAGTATCCGCCATGGACATCCCTCGACTTTGCATCTGTGGTGGGCTCGCCGTCCTCTGGCCGCAGCCCGCGCAGTAATTTTCGCTCAGATGGTAAATGACCCTGGATATCAACAGGGCGGTGGTTTTCGTTACGGTGTCAACAAAGAGAAAGCACAACTGGAACGTGAACGTTTGTTCAAAATTATCGAAGAACTTGTTCAGTGGGAAAACACTAACAACGAGGCGGTCCTTTCGCGCGCGCGTGCTGAAATCTGGAAGTCTTGGCGTGAGACTTGCGAGTTGAATAAAAACCATCCGTGTGCAGCCGAACTGTTCAACCCAGATAAACTTCCTGCCTTTCACGACCCATTCGCGGGAGGGGGAGCCATTCCATTGGAGGCTCAACGCCTTGGATTAGAATCATATGCCTCTGACCTGAATCCTGTCGCCGTAACCATTAATAAGGCGATGATTGAGATCCCTCCTCGCTTTGCCGGTCGCGCGCCGGTGGGTCCTGTACCTCCCAGCCCGGACGGGCGTGGGGTTGGCGGGGAAGGTTTATTCGCTCAGGACTGGGCAGGAGCAAAAGGTCTGGCCGAAGATGTTCGCCGCTATGGAGCGTGGATGCGTTCCGAAGCAGAGAAGCGTATTGGCCATCTTTATCCGCAGGTCGAAGTAACTCGCGAGTTGGCTCAGGGACGCCAAGATCTGCAACCCTTAGTTGGACAAAAATTGACGGTAATCGCATGGCTGTGGGCGCGCACGGTAAAATCTCCCAACCCGGCTTTTTCGCATGTAGAGGTTCCGTTGGCTTCGACGTTCGTCTTGTCGTCGAAAGCTGGCAAGGAGGCTTACGTCCAGCCGATGATCTCGCCTCTTCCTTTAGGTGAAGGACTTGGAGTCCGCGCAGGCTCCGAGGGATATTACCGTTTCACAGTCCAGGTTGCGGGTACGCCAGGTTTCGACAAAGCCGATTATGCCCGTGCGAAATCGGGAACGAAACTGGCACGCGGTGCTAATTTCGAGTGCCTTCTGTCAAATACACCGATTGAACCCAACCATATTTACACCGAAGCTAATGCCGGACGCATGGGAGCGCGCCTGATGGCAATCGTAGCAGAGGGTGCGCGCGGTCGCGTATATTTGCCCCCCCTGCCAGAACATGAAGCGATTGCTCGTCAGGCACAGCCTGAGTGGAAGCCGGAGGTAGCCATGCCTGATAATCCCCGTTGGTTCTCGCCCCCTTTGTACGGATTAAAAAATTATGGGGACTTATTTACCCCTCGTCAGTTGGTTGCGTTAACAACTTTCTCAGATCTTGTTATCGATGCCATTGAGCGTTGTCGTCGTGATGCTGCAGCGGCGGGACTTCCTGATGATGGCGTCCCCTTGGATGCCGGAGGGACCGGAGCGACTGCGTACGCACAGGCGGTTGGCGTCTACTTAGCAATCGCTATCTCTCGTTTTTCGGACCGCAACAACAGTATCTGTACGTGGGATTCGGGACCAACCGGGACAAAGGCTAGTACAGGTGGTTCAGCACGTACTGCGTCTCTTCGCAATTTGTTCGCTCGTCAAGCCATCCCGATGGCCTGGGATTTTGGTGAGGCTAACCCGTTTAGCGACTCGGGTGGGGGTTTCAGTAGTGCTTTTGAATGGATCGAACCTGCGGTCCGCTCCTTACGTGGGGGATGTGCAGGGTATGGTGACGGGGCTGACGCACAGACACAGACGCTGTCACGTGACAAAGTGGTCAGCACCGACCCACCCTACTATGACAACATCGGCTACGCTGACCTGTCAGACTTTTTCTACGTCTGGCTGCGCCGCAGCTTGAAGCCCATCTTCCCAGGACTTTATGCAACCTTGGCAGTACCAAAGGCCGAGGAACTGGTCGCAACACCCTATCGCCATGGCTCCAAGGAAGCAGCGGAGGCGTTCTTTCTGGACGGAATGCGTCGCGCATTAAAGAATCTTGCCGAGCAGGCGCATCCTGCCTTTCCAGTAACTATTTATTACGCGTTCAAGCAGTCAGAGACTACAGACGCGGCGGGAACCTCTTCGACGGGATGGGAGACTTTCCTTCAGGCTGTCCTTGATGCTGGCTTTGCATTGACGGGAACATGGCCGATGCGCACAGAATTGGGCAATCGCATGATTGGAGCTGGTACGAATGCATTGGCTAGCAGTATCGTCTTGGTTTGTCGTCAACGTGCCACCGATGCTCCCACCGCAAGCCGTCGCGAATTCTTACGTGAATTGAATGCTACTCTTCCAGAGGCTATCGCAGACATGATTGGGGCAGATCCCAGTCCTCAACCTCTTTCTCCGCGCGAGCGTGGGTATGGACGCGTGGCTCCTGTGGACCTTAGCCAAGCTATCATTGGACCTGGCATGGCGATTTTTTCCCAGTATGCGGCGGTATTGGAGGCTGATGGGACACCGATGACCGTCAAGACTGCTTTGGCATTGATCAATCGCTTTCTTGCGGAAGATGATTTCGATCACGATACGCAATTTTGTTTGCATTGGTTTGAGCAACAGGGTTGGGCATCTGGTAAATATGGAGAGGCCGACGTCTTGGCGCGTGCAAAGGGGACCGCGGTTGATGCGCTTGTCGCAGCTGGTGTTGCTGAGAGCGCTAAAGGAAGCGTTCGTTTGCTGAAGTGGCCGGAATACCCTGCCGATTGGTCTCCTGAAAGTGACACTCGCACACCCATTTGGGAAGCATTACACCAATTGATCCGCGCACTTAACCAAGCGGGGGAGACTGAAGCTGGCCGCTTATTGGCTCGCATGCCTGCCCGCGCTGAGCCTATCCGCGCTCTTGCTTATCGTTTATATACTCTGTGTGAGCGTAAAGGGTGGGCGGAGGACGCTCGTGCGTATAATGAGCTTGTGACGGCGTGGTCAGGGATCGAGCAGGCCGCGAATGAAGCAGGTGTGGTCGGCGCACAGATGCAGTTAGAGCTGTGA |

**Tables S7. BlastP analysis.** Excel file
